# Forensic Gender Determination by Using Mandibular Morphometric Indices an Iranian Population: A Panoramic Radiographic Cross-Sectional Study

**DOI:** 10.3390/jimaging9020040

**Published:** 2023-02-11

**Authors:** Mahsa Esfehani, Melika Ghasemi, Amirhassan Katiraee, Maryam Tofangchiha, Ahad Alizadeh, Farnaz Taghavi-Damghani, Luca Testarelli, Rodolfo Reda

**Affiliations:** 1 Department of Oral and Maxillofacial Medicine, Dental Caries Prevention Research Center, Qazvin University of Medical Sciences, Qazvin 34199-15315, Iran; 2 Student Research Committee, Qazvin University of Medical Sciences, Qazvin 34199-15315, Iran; 3 Department of Orthodontics, Dental School, Shahed University of Medical Sciences, Tehran 33191-18651, Iran; 4 Department of Oral and Maxillofacial Radiology, Dental Caries Prevention Research Center, Qazvin University of Medical Sciences, Qazvin 34199-15315, Iran; 5 Medical Microbiology Research Center, Qazvin University of Medical Sciences, Qazvin 34199-15315, Iran; 6 Department of Prosthodontics, Dental Caries Prevention Research Center, Qazvin University of Medical Sciences, Qazvin 34199-15315, Iran; 7 Department of Oral and Maxillo-Facial Science, Sapienza University of Rome, 00161 Rome, Italy

**Keywords:** mandible, radiography, panoramic, gender determination, forensic anthropology

## Abstract

Gender determination is the first step in forensic identification, followed by age and height determination, which are both affected by gender. This study assessed the accuracy of gender estimation using mandibular morphometric indices on panoramic radiographs of an Iranian population. This retrospective study evaluated 290 panoramic radiographs (145 males and 145 females). The maximum and minimum ramus width, coronoid height, condylar height, antegonial angle, antegonial depth, gonial angle, and the superior border of mental foramen were bilaterally measured as well as bicondylar and bigonial breadths using Scanora Lite. Correlation of parameters with gender was analyzed by univariate, multiple, and best models. All indices except for gonial angle were significantly different between males and females and can be used for gender determination according to univariate model. Condylar height, coronoid height, and superior border of mental foramen and ramus were still significantly greater in males than in females after controlling for the effect of confounders (*p* < 0.05). Based on the best model, a formula including five indices of bicondylar breadth, condylar height, coronoid height, minimum ramus width, and superior border of mental foramen was used for gender determination. Values higher than 56% indicate male gender, while lower values indicate female gender, with 81.38% specificity for correct detection of females and 88.97% sensitivity for correct detection of males. Despite the satisfactory results, future research should focus on larger populations to verify the accuracy of the present findings.

## 1. Introduction

In accidents and disasters with a high number of fatalities, identification of victims is highly important. This process would be simple and could be done with 100% certainty if the bodies are intact [1,2]. However, in accidents and disasters such as a plane crash, flood, or earthquake, the bodies may be severely damaged, making their identification almost impossible. Forensic gender determination is the first step in such cases, followed by age determination and height determination, which are both affected by gender [3]. Accurate forensic identification depends on the presence of intact remains; in that case, correct identification can be done with up to 95% certainty [4,5,6].

Human bones mostly differ in size between males and females. According to a recent review conducted in 2022, the greatest geometric morphometric difference between males and females is in the pelvis, followed by the humerus and the cranium [7]. If the cranium is not available for any reason, or is severely damaged, the mandible can be used for forensic gender determination since it has the greatest difference between males and females [8]. It is also the largest and the hardest bone of the skull [1,8].

Panoramic radiography was introduced in 1950, and since then, it is among the most commonly used imaging modalities. Panoramic radiography is a simple extraoral radiography that visualizes the mandible, maxilla, temporomandibular joint, and related structures all in one radiograph [9,10,11,12].

Several mandibular morphometric indices have been proposed for forensic gender determination, such as the bicondylar breadth, intercoronoid distance, and some other vertical and horizontal distances measurable on panoramic radiographs [13]. The mental foramen and its vertical and horizontal distance from the borders of the mandible can also be used for the purpose of gender determination [14,15].

Moreover, the commonly used 3D diagnostic modalities, and the increasing photographic information that can be matched with these examinations depending on the chosen landmarks, offer even more possible solutions [16].

Undeniably, gender determination according to bone remains is highly important in forensic anthropology [17]. Considering the wide variation in indices used for this purpose, finding the most reliable and reproducible indices is imperative. In addition, racial and ethnic parameters may affect the reliability of indices in different populations. Thus, this study aimed to find the most reliable indices for forensic gender determination and assess their accuracy on panoramic radiographs of an Iranian population.

## 2. Materials and Methods

This retrospective study was conducted on 290 panoramic radiographs of 145 males and 145 females between 18 and 70 years, retrieved from the archives of a radiology clinic in Qazvin, Iran. The study was approved by the ethics committee of Qazvin University of Medical Sciences (IR.QUMS.REC.1400.391).

### 2.1. Sample Size

The sample size was calculated using the following formula, where alpha = 0.05, N_1_ = 145, N_2_ = 145, µ_1_ = 109.74, µ_2_ = 113.36, σ_1_ = 12.24, and σ_2_ = 9.51.

$$n=\frac{\left(\sigma_{1}^{2}+\sigma_{2}^{2}\right){\left(z_{1-\frac{\propto }{2}}+z_{1-\beta }\right)}^{2}}{\Delta^{2}}$$

### 2.2. Eligibility Criteria

The inclusion criteria were (I) panoramic radiographs taken between January 2022 and May 2022 in one radiology clinic for purposes not related to this study, and (II) the availability of demographic information of patients.

The exclusion criteria were (I) incomplete visualization of the right or left side on the radiograph, (II) absence of the inferior border of the mandible on the radiograph, (III) absence or poor visualization of the mental foramen on the radiograph, (IV) absence of bilateral symmetry, and (V) presence of distortion.

All panoramic radiographs had been taken using Rayscan alpha scanner (Ray Co., Ltd., Hwaseong-si, Republic of Korea) with an active exposure voltage between 62 and 80 kV, current range between 10–14 mA, and time of 13.5 s.

**Figure 1:**
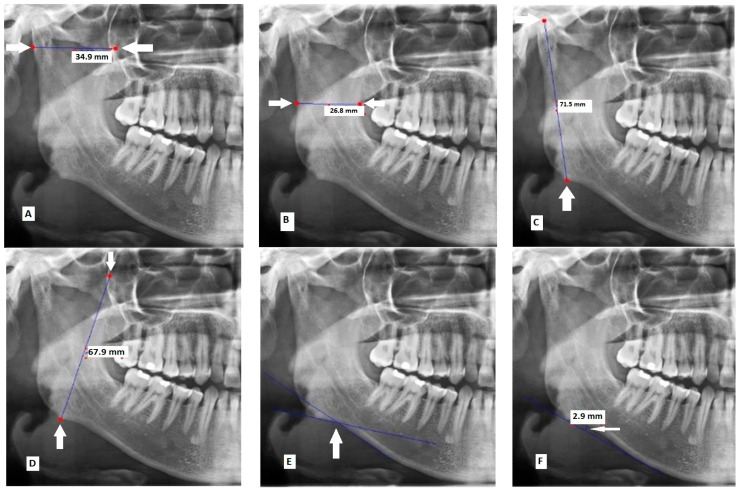

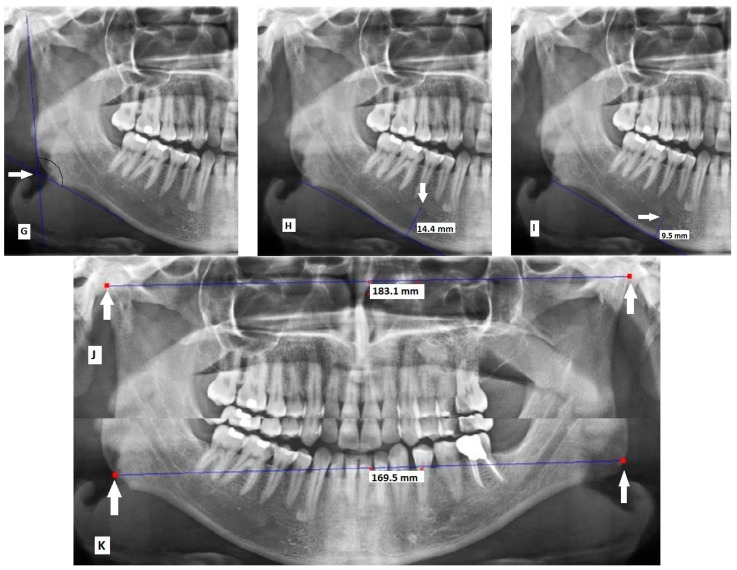
Measurements made on panoramic radiographs: (**A**) maximum ramus width; (**B**) minimum ramus width; (**C**) condylar height; (**D**) coronoid height; (**E**) antegonial angle; (**F**) antegonial depth; (**G**) gonial angle; (**H**) distance between the superior border of the mental foreman and the inferior border of the ramus; (**I**) distance between the inferior border of the mental foramen and the inferior border of the ramus; (**J**) bicondylar breadth; and (**K**) bigonial breadth.

The images were assessed using Scanora (version 5.0.2) software (Digora, Helsinki, Finland) [18]. The following 9 parameters were measured in millimeters, bilaterally:

- Maximum ramus width: Maximum anteroposterior ramus width (Figure 1A) [16].
- Minimum ramus width: Minimum anteroposterior ramus width (Figure 1B) [16].
- Condylar height: Distance between the most superior part of the condyle and the most inferior part of the inferior border of the body of mandible (Figure 1C) [19].
- Coronoid height: Distance between the most superior part of the coronoid process and the most inferior part of the inferior border of the body of mandible (Figure 1D) [20].
- Antegonial angle: Intersection of two lines at the deepest point of the inferior border of mandible. The first line passes through the anterior part of the inferior border of mandible and the second line passes through the inferior border at the gonion (Figure 1E) [21].
- Antegonial depth: Vertical distance between the inferior border of the mandible and its deepest point (Figure 1F) [21].
- Gonial angle: Angle formed between the following two lines: A line tangent to the ramus and the mandibular condyle, and a line tangent to the most inferior part of the gonial section and the body of the mandible (Figure 1G) [18].
- Distance between the superior border of the mental foramen and the inferior border of the ramus (Figure 1H) [22].
- Distance between the inferior border of the mental foramen and the inferior border of the ramus (Figure 1I) [23].

The following two parameters were also measured in millimeters:

- Bicondylar breadth: distance between the most posterior part of the posterior border of the right and left condyles (Figure 1J) [20].
- Bigonial breadth: distance between the right and left gonia (Figure 1K) [18].

The anatomical landmarks for the measurements were first identified by an oral medicine specialist and an oral and maxillofacial radiologist, with 95% interexaminer agreement [24]. If the two observers did not agree on the location of any anatomical landmark, an experienced oral and maxillofacial radiologist would guide them to reach a consensus. Next, the parameters were measured digitally by a trained senior dental student using Scanora (version 5.0.2) software (Digora, Helsinki, Finland).

### 2.3. Statistical Analysis

Data were analyzed using R software (version 4.2.2) [25]. Three analytical models were applied. Each analysis was conducted for three groups of indices: right, left, and mean of both sides. First, univariate logistic regression was independently performed for each parameter without considering the effect of other indices. Next, multiple logistic regression was applied to consider the possible confounding effects of other indices of the mandible. Best model was finally applied to find the most reliable predictors for forensic gender determination according to the Akaike information criterion, which is a criterion for model selection using MASS package in R [26]. The plots were drawn by ggplots2 [27]. The “verification” package of R was used to evaluate the accuracy of indices and estimate their threshold, specificity, and sensitivity [28].

## 3. Results

**Figure 2:**
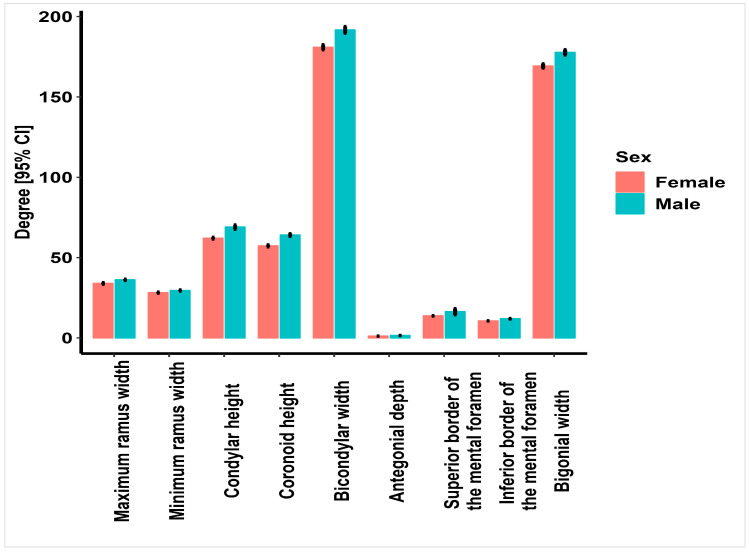
Mean values (mm) of the 9 indices measured bilaterally in males and females.

A total of 290 panoramic radiographs of 145 males and 145 females between 18 and 70 years were evaluated. Figure 2 shows the mean values of the nine indices measured bilaterally in males and females.

**Figure 3:**
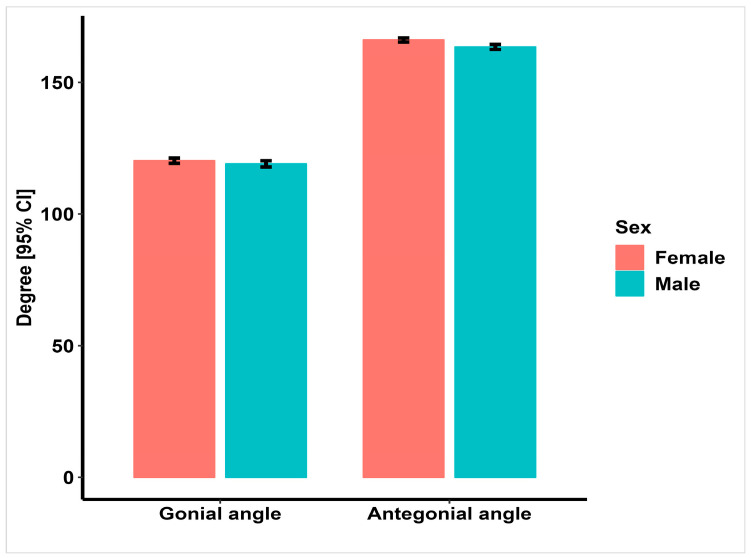
Mean values (degree) of gonial and antegonial angles in males and females.

Figure 3 shows the mean values of the gonial and antegonial angles in males and females.

The univariate analysis, independently performed for each factor without the effect of other indices, revealed that all indices, except for gonial angle, were significantly different between males and females (*p* < 0.05). Except for antegonial angle, which was significantly larger in females, all other indices were significantly greater in males (*p* < 0.05).

Multiple model was then applied, considering the possible effect size of other indices of the mandible. Different results were obtained by multiple analysis, compared with univariate analysis, after controlling for the effect of different variables. Condylar height, coronoid height, and distance between the superior border of the mental foramen and the border of the ramus were significantly greater in males than in females in this analysis (*p* < 0.05). However, no difference was found in other variables (*p* > 0.05).

The best-fit model was finally applied, and the mean of the bilateral antegonial depth, bicondylar breadth, coronoid height, condylar height, minimum ramus width, and distance between the inferior and superior borders of the mental foramen and the border of mandible were entered into the analysis (Table 1).

Best-model analysis was conducted by designing a formula. Accordingly, a formula was proposed with five indices of bicondylar breadth, condylar height, coronoid height, minimum ramus width, and distance between the superior border of the mental foramen and the inferior border of the mandible for forensic gender determination, and the threshold for gender determination was found to be 0.56. Accordingly, higher and lower values of this threshold indicated male and female gender, respectively. This test had an 81.38% sensitivity (73.793–88.966) (correct detection of males) and an 88.97% specificity (correct detection of females). This threshold was 0.51 for the right side and 0.58 for the left side.

**Figure 4:**
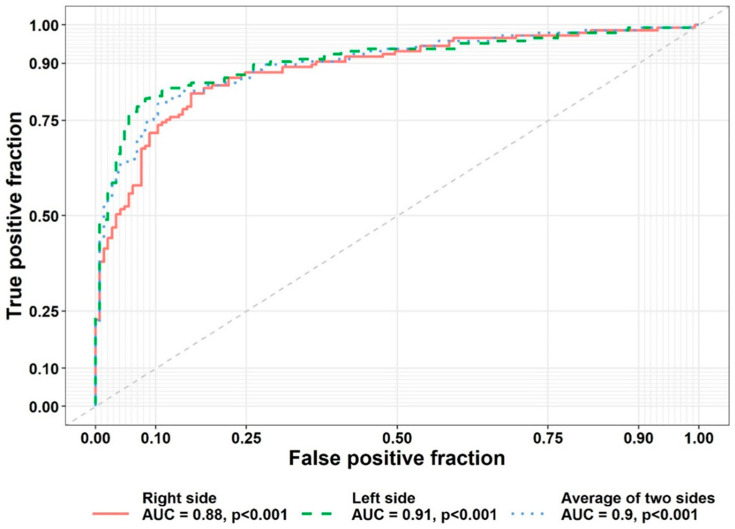
Receiver operating characteristic curve for sensitivity of the formula.

Figure 4 presents the receiver operating characteristic curve for the sensitivity of this test.

Table 2 presents the sensitivity and specificity of indices on the right and left sides and the mean of both sides for gender determination. According to the results, the most reliable indices for gender determination were found to be condylar height and coronoid height, with probabilities of 87.4% and 82.7%, respectively, while the gonial angle had the lowest probability of 55.5%.

## 4. Discussion

This study assessed forensic gender determination by using morphometric indices of the mandible on panoramic radiographs of an Iranian population. Eleven mandibular indices were evaluated, out of which nine were bilateral. The results showed that in bilateral assessment with the best model, five indices could be effectively used for forensic gender determination, including bicondylar breadth, condylar height, coronoid height, minimum ramus width, and distance between the superior border of the mental foramen and the inferior border of the mandible. In the same vein, univariate analysis of the indices revealed that all of them had a significant role in gender determination, except for gonial angle.

The multiple model showed that, bilaterally, condylar height, coronoid height, and distance between the superior border of the mental foramen and the inferior border of the mandible were significantly different in males and females and could be used for forensic gender determination.

A few studies are available regarding some of these indices, such as the study by Iliescu et al. [23], who assessed several linear, angular, and relative parameters in the mandible and showed their high potential for gender determination. Rad et al. [18] evaluated some of these factors in an Iranian population and found that all of them were significantly different between males and females, including the gonial angle. They reported that ramus height, bicondylar breadth, and coronoid height were the most accurate parameters for gender determination. They suggested a formula with four variables of right ramus height, chin height, bicondylar breadth, and right coronoid height. However, in the present study, the variables were assessed in three different states: only the right side, only the left side, and the mean of both sides. Five factors were found to have a significant role in gender determination. However, Rad et al. [18] reported the accuracy of gender determination to be 82.5% according to the right-side parameters and 82.9% according to the left-side parameters, which were slightly lower than the values obtained in the present study. Dabaghi and Bagheri [20] assessed coronoid height, ramus height, mandibular body height, and bicondylar breadth. They concluded that all of them were significantly different in males and females and that gender determination could be performed with 89% accuracy by measuring these four factors. Ulusoy and Ozkara [29] found that all the measured variables, including condylar height, coronoid height, minimum and maximum ramus width, bigonial breadth, bicondylar breadth, and mental foramen height, were significantly larger in males than females in univariate analysis, and only the gonial angle was not significantly different. The findings of the present study were consistent with their results.

However, a noteworthy issue is that only part of the mandible may be available for gender determination. Thus, in the present study, all indices were evaluated bilaterally (mean of both sides) and also separately on each side. On the right side, the best model showed that condylar height, coronoid height, minimum ramus width, and vertical distance between the inferior and superior borders of mental foramen and ramus border all had a significant role in gender determination. Significant indices on the left side were antegonial depth, condylar height, and vertical height of the superior border of the mental foramen. For each side, a threshold was defined according to the best model. On the right side, sensitivity (correct detection of males) and specificity (correct detection of females) were 82.76% and 84.83%, respectively. These values were 82.07% and 91.72% on the left side, respectively.

In the next step, each index was separately assessed, and sensitivity and specificity values were reported. The results showed that condylar height, coronoid height, and the superior and inferior borders of the mental foramen had the highest accuracy for gender determination.

### 4.1. Antegonial Angle and Antegonial Depth

According to univariate analysis, the antegonial angle was significantly smaller in males than in females. Unlike other factors, by an increase in size of this angle, the possibility of female gender increased. The gender determination threshold was 163.75 for this parameter. If the obtained value is lower than this threshold, the possibility of female gender would be 63%. The size of this angle in the right side was slightly larger than the left side. Gender determination by this method had a relatively low sensitivity of 50.34%; however, specificity was 73.1%. The results of Ghosh et al. [30], Tozoğlu and Çakur [31], and Dutra et al. [32] approved the present findings. Due to the inverse effect of antegonial angle on antegonial depth, as reported in the literature, antegonial depth in males is larger than in females. In the present study, the antegonial depth was significantly larger in males than in females, and its threshold for gender determination was 1.08 cm. The possibility of correct gender determination by this index was approximately similar to that by the antegonial angle (62.7%).

### 4.2. Bicondylar Breadth

In univariate and best-model analyses, this index was significantly larger in males than in females. Gender determination threshold was 185.2 mm, with a relatively high sensitivity and specificity of 71.03% and 71.72%, respectively. The possibility of correct gender determination by this index was 74.7%. El-Shafey et al. [33] and Kharoshah et al. [34] in Egypt reported similar results. Two studies conducted in Iran by Dabaghi and Bagh [20] and Rad et al. [18] reported the same results as well. In the study by Rad et al. [18], bicondylar breadth was among the four indices with maximum accuracy for gender determination.

### 4.3. Bigonial Breadth

Bigonial breadth showed high probability for gender determination (71%). According to univariate analysis, this index in males was significantly larger than that in females. The specificity of this index was higher than its sensitivity, which means that it determines female gender with 80% probability and male gender with 58% probability. Similar results were observed by Leversha et al. [35] in an Australian population, El-Shafey et al. [33] in an Egyptian population, and Rad et al. [18] in an Iranian population. However, this parameter had a higher percentage of accuracy for gender determination in the present study.

### 4.4. Condylar Height

This parameter had the highest accuracy and probability for gender determination in the present study. All three analyses of univariate, multiple, and best-model showed a significantly higher condylar height in males than in females, and this parameter, with 87% probability and high sensitivity and specificity, can correctly determine gender. Previous studies have also confirmed this finding [19,20,36]. The threshold for this parameter was 66.72 mm, such that higher values would indicate male gender and vice versa.

### 4.5. Coronoid Height

After condylar height, coronoid height was found to be the most reliable index for gender determination in the present study. All three analyses showed correct gender determination by this parameter with 82.65% probability, and high sensitivity and specificity, which is in accordance with previous findings [19,20,36,37]. In addition, it is among the indices used in the best-model formula for gender determination [18]. In the present study, the coronoid height threshold for gender determination was 61.38 mm, which was almost in agreement with previous studies. Slight differences can be attributed to the type of scanner and software.

### 4.6. Gonial Angle

Gonial angle was the only parameter that was not suitable for gender determination, neither in univariate or in multiple analyses on any side, nor in the mean value of both sides in the present study. Although this parameter was slightly greater in males than in females, this difference was not statistically significant. The results of previous studies in this regard have been controversial. For example, Saleh et al. [38] found no significant difference in this parameter between males and females, which was the same as the present finding. However, Rad et al. [18] in Iran and Sandeepa et al. [37] in Saudi Arabia showed that the gonial angle of males was significantly larger than that of females; however, they did not include it in best model for gender determination in their study.

### 4.7. Superior and Inferior Borders of Mental Foramen

In the present study, the position of the mental foramen was vertically assessed relative to the inferior border of the mandible. The univariate analysis showed that it can be used for gender determination with 75% probability for its inferior border and 78% probability for its superior border. This parameter had high specificity but lower sensitivity. In other words, it was more accurate for the detection of females than males. This parameter is among the anatomical landmarks that remain constant throughout life and their position does not change with age. Thus, it can be used for gender determination at any age. The results of previous studies in this respect were in accordance with the present findings [22,39,40,41].

### 4.8. Maximum and Minimum Ramus Width

Maximum ramus width was reliable for gender determination according to univariate but not multivariate analysis. However, minimum ramus width in both models, as well as the best model, was significantly different in males and females. These parameters can be used for gender determination but do not have high sensitivity and specificity. Similar results have been reported in the literature [19,33,37].

Although studies similar to the present one have been previously conducted in different populations, this study included a high number of parameters and used the best model to account for the interaction effect of different parameters and obtain more reliable results. However, further studies are required to verify the accuracy of the present findings in a larger population.

This study was conducted only on the population of Qazvin city, Iran. Thus, the results may not be generalizable to the entire population of Iran. In addition, differences in scanners and software programs can affect the results, which should be taken into account in comparison of results with the available literature. Further studies can be conducted on other populations with larger sample sizes for identification of racial and ethnic differences through the mandible.

## 5. Conclusions

According to the univariate model, all indices, bilaterally, except for gonial angle, can be used for gender determination. According to the best model and by adjusting for the effect size of indices on each other, a formula was proposed with five indices of bicondylar breadth, condylar height, coronoid height, minimum ramus width, and distance between the superior border of the mental foramen and inferior border of the mandible for gender determination. Values > 0.56 would indicate male gender, while lower values would indicate female gender, with 81.38% specificity for correct detection of females and 88.97% sensitivity for correct detection of males.

## Figures and Tables

**Table 1:** Results obtained in univariate, multiple, and best models.

***Right***						
	**Multiple model**		**Univariate model**		**Best model**	
**Index**	OR (95% CI)	*p* value	OR (95% CI)	*p* value	OR (95% CI)	*p* value
**AGA**	0.98 (0.89, 1.07)	0.624	0.91 (0.86, 0.95)	<0.001 ***		
**AGD**	1.33 (0.75, 2.34)	0.323	1.66 (1.26, 2.22)	<0.001 ***	1.37 (0.95, 2.01)	0.092
**BCW**	1.04 (1, 1.09)	0.077	1.08 (1.06, 1.11)	<0.001 ***	1.06 (1.03, 1.1)	<0.001
**BGW**	1.04 (0.98, 1.1)	0.183	1.09 (1.06, 1.12)	<0.001 ***		
**CH**	1.07 (1.03, 1.14)	0.003	1.26 (1.19, 1.33)	<0.001 ***	1.08 (1.03, 1.14)	0.002
**CRH**	1.17 (1.07, 1.28)	0.001	1.29 (1.21, 1.37)	<0.001 ***	1.16 (1.08, 1.26)	<0.001
**GA**	1.03 (0.97, 1.1)	0.328	0.97 (0.94, 1.01)	0.135		
**IBMF**	0.59 (0.34, 1.01)	0.059	1.84 (1.54, 2.23)	<0.001 ***	0.58 (0.33, 0.99)	0.049
**SBMF**	2.31 (1.36, 4.04)	0.002	2.02 (1.68, 2.47)	<0.001 ***	2.36 (1.39, 4.1)	0.002
**MaxRW**	1.01 (0.83, 1.18)	0.941	1.22 (1.13, 1.32)	<0.001 ***		
**MinRW**	0.84 (0.67, 1.05)	0.129	1.19 (1.09, 1.3)	<0.001 ***	0.86 (0.75, 0.97)	0.021
***Left***						
	**Multiple model**		**Univariate model**		**Best model**	
**AGA**	1.01 (0.91, 1.12)	0.789	0.89 (0.84, 0.94)	<0.001 ***		
**AGD**	1.66 (0.87, 3.16)	0.122	1.78 (1.34, 2.41)	<0.001 ***	1.55 (1.02, 2.42)	0.047
**BCW**	1.04 (0.99, 1.09)	0.112	1.08 (1.06, 1.11)	<0.001 ***	1.04 (1, 1.08)	0.031
**BGW**	1.01 (0.95, 1.06)	0.843	1.09 (1.06, 1.12)	<0.001 ***		
**CH**	1.31 (1.18, 1.48)	<0.001	1.41 (1.31, 1.53)	<0.001 ***	1.31 (1.18, 1.46)	<0.001
**CRH**	1.07 (0.96, 1.19)	0.216	1.29 (1.22, 1.38)	<0.001 ***	1.08 (0.98, 1.19)	0.135
**GA**	1.05 (0.99, 1.13)	0.13	0.97 (0.94, 1.01)	0.128	1.05 (0.99, 1.12)	0.108
**IBMF**	0.88 (0.49, 1.55)	0.664	1.98 (1.63, 2.43)	<0.001 ***		
**SBMF**	1.51 (0.88, 2.69)	0.145	2.09 (1.73, 2.58)	<0.001 ***	1.36 (1.06, 1.77)	0.02
**MaxRW**	1.01 (0.85, 1.18)	0.898	1.22 (1.13, 1.32)	<0.001 ***		
**MinRW**	0.87 (0.71, 1.04)	0.141	1.18 (1.08, 1.28)	<0.001 ***	0.88 (0.76, 1.01)	0.084
***Total (mean of left and right)***						
	**Multiple model**		**Univariate model**		**Best model**	
**AGA**	0.99 (0.89, 1.09)	0.847	0.9 (0.85, 0.94)	<0.001 ***	-	-
**AGD**	1.4 (0.76, 2.56)	0.281	1.73 (1.3, 2.33)	<0.001 ***	1.37 (0.93, 2.06)	0.122
**BCW**	1.04 (1, 1.09)	0.059	1.08 (1.06, 1.11)	<0.001 ***	1.06 (1.03, 1.1)	<0.001
**BGW**	1.03 (0.97, 1.09)	0.382	1.09 (1.06, 1.12)	<0.001 ***	-	-
**CH**	1.16 (1.08, 1.27)	<0.001	1.34 (1.25, 1.43)	<0.001 ***	1.17 (1.09, 1.27)	<0.001
**CRH**	1.13 (1.02, 1.25)	0.02	1.3 (1.22, 1.38)	<0.001 ***	1.11 (1.02, 1.22)	0.014
**GA**	1.04 (0.97, 1.11)	0.283	0.97 (0.94, 1.01)	0.129	-	-
**IBMF**	0.57 (0.29, 1.12)	0.109	2.08 (1.71, 2.6)	<0.001 ***	0.54 (0.27, 1.05)	0.074
**SBMF**	2.48 (1.29, 4.96)	0.008	2.25 (1.83, 2.81)	<0.001 ***	2.64 (1.38, 5.27)	0.004
**MaxRW**	1 (0.81, 1.19)	0.985	1.24 (1.14, 1.35)	<0.001 ***	-	-
**MinRW**	0.84 (0.66, 1.05)	0.134	1.2 (1.1, 1.31)	<0.001 ***	0.84 (0.72, 0.96)	0.013

*** Statistically significant.

**Table 2:** Sensitivity and specificity of indices in the right and left sides and the mean of both sides for gender determination.

		Threshold	Specificity (%)	Sensitivity (%)	A	*p* Value	Direction
**AGA**	Right	164.5 (159.5, 169.5)	65.52 (21.379, 93.103)	55.86 (23.448, 93.103)	0.6207	<0.001 ***	indirect
	Left	163.5 (161.5, 168.5)	78.62 (34.483, 89.655)	46.21 (31.034, 85.517)	0.6449	<0.001 ***	indirect
	Total	163.75 (161.75, 168.25)	73.1 (37.241, 90.345)	50.34 (28.966, 82.759)	0.6339	<0.001 ***	indirect
**AGD**	Right	1.05 (0.55, 2.25)	59.31 (31.034, 93.103)	64.14 (24.138, 86.914)	0.62	<0.001 ***	direct
	Left	1.15 (0.75, 1.95)	63.45 (44.138, 88.966)	61.38 (30.345, 77.241)	0.6316	<0.001 ***	direct
	Total	1.08 (0.9, 1.925)	62.07 (45.517, 88.966)	63.45 (31.724, 77.931)	0.6276	<0.001 ***	direct
**BCW**		185.2 (180.45, 191.9)	71.72 (53.103, 89.655)	71.03 (49.655, 87.586)	0.747	<0.001 ***	direct
**BGW**		175.95 (172.9, 180.85)	80 (66.897, 94.483)	57.93 (39.31, 70.345)	0.7171	<0.001 ***	direct
**CH**	Right	66.7 (66.3, 67.85)	92.41 (86.897, 97.931)	77.93 (68.966, 84.828)	0.8751	<0.001 ***	direct
	Left	66.8 (64.85, 67.35)	93.1 (84.828, 97.241)	77.93 (69.655, 86.207)	0.8822	<0.001 ***	direct
	Total	66.72 (65.625, 67.125)	93.79 (86.897, 97.241)	77.93 (70.345, 85.517)	**0.8745**	<0.001 ***	direct
**CRH**	Right	61.75 (60.45, 62.25)	85.52 (76.552, 92.414)	72.41 (62.759, 81.379)	0.8202	<0.001 ***	direct
	Left	61.35 (60.45, 62.45)	86.21 (78.621, 92.414)	69.66 (61.379, 78.621)	0.8238	<0.001 ***	direct
	Total	61.38 (60.075, 62.275)	86.21 (77.931, 92.414)	72.41 (63.431, 80)	**0.8265**	<0.001 ***	direct
**GA**	Right	119.5 (110.5, 124.5)	64.83 (28.966, 96.552)	50.34 (12.414, 82.759)	0.5545	0.0542	indirect
	Left	118.5 (109.5, 122.5)	66.9 (33.103, 97.931)	48.97 (10.345, 79.31)	0.556	0.0493	indirect
	Total	118.75 (110.25, 123.75)	66.21 (32.414, 97.931)	50.34 (12.414, 80.69)	**0.5553**	0.0516	indirect
**SBMF**	Right	15.35 (14.45, 15.55)	89.66 (74.483, 95.172)	57.24 (47.586, 71.724)	0.7679	<0.001 ***	direct
	Left	14.45 (14.05, 15.65)	66.9 (52.414, 90.345)	78.62 (50.345, 89.655)	0.7752	<0.001 ***	direct
	Total	15.15 (13.925, 15.35)	86.21 (56.552, 93.103)	62.76 (52.414, 88.966)	0.7871	<0.001 ***	direct
**IBMF**	Right	11.55 (11.15, 12.55)	77.24 (65.517, 97.241)	62.07 (35.862, 73.793)	0.7278	<0.001 ***	direct
	Left	11.15 (10.95, 11.65)	71.72 (60.69, 86.207)	71.72 (55.172, 82.069)	0.7502	<0.001 ***	direct
	Total	11.72 (11.075, 11.975)	84.14 (66.897, 91.034)	60.69 (49.655, 77.241)	0.7516	<0.001 ***	direct
**Max RW**	Right	35.05 (33.75, 36.45)	67.59 (51.034, 83.448)	71.72 (53.793, 85.517)	0.7083	<0.001 ***	direct
	Left	35.2 (33.55, 36.95)	66.9 (44.828, 89.655)	68.97 (42.759, 88.276)	0.7077	<0.001 ***	direct
	Total	35.02 (33.3, 36.075)	65.52 (48.276, 79.31)	73.1 (57.241, 86.897)	0.7144	<0.001 ***	direct
**Min RW**	Right	29.25 (26.85, 31.15)	66.9 (33.793, 91.724)	58.62 (29.655, 88.966)	0.6352	<0.001 ***	direct
	Left	29.75 (26.45, 31.25)	72.41 (29.655, 88.966)	53.79 (33.103, 92.414)	0.642	<0.001 ***	direct
	Total	29.58 (26.725, 31.05)	68.97 (32.414, 91.034)	57.24 (31.724, 90.345)	0.6416	<0.001 ***	direct

*** Statistically significant.

## Data Availability

Not applicable.
